# Spotted Fever Rickettsioses in Panama: New Cases and the Gaps That Hinder Its Epidemiological Understanding

**DOI:** 10.3390/pathogens14101006

**Published:** 2025-10-04

**Authors:** Sergio Bermúdez, Ericka Ferguson Amores, Naty Aguirre, Michelle Hernández, Boris Garrido, Lillian Domínguez, Yamitzel Zaldívar, Claudia González, Jorge Omar Castillo, Alexander Martínez-Caballero, Ambar Moreno, Mabel Martínez-Montero, Ambar Poveda, Domicio Espino, Karina Baker, Franklyn Samudio

**Affiliations:** 1 Instituto Conmemorativo Gorgas de Estudios de la Salud, Ciudad de Panamá, Panama; mihernandez@gorgas.gob.pa (M.H.); ldominguez@gorgas.gob.pa (L.D.); yzaldivar@gorgas.gob.pa (Y.Z.); cgonzalez@gorgas.gob.pa (C.G.); almartinez@gorgas.gob.pa (A.M.-C.); ammoreno@gorgas.gob.pa (A.M.); mcmartinez@gorgas.gob.pa (M.M.-M.); fsamudio@gorgas.gob.pa (F.S.); 2 Hospital Materno Infantil “José Domingo De Obaldía”, David, Panama; jorge.castillo@hobaldia.sld.pa (J.O.C.);; 3 Hospital “Aquilino Tejera”, Penonomé, Panama; nlaguirren@minsa.gob.pa (N.A.); prax_27@yahoo.com (D.E.); 4 Centro de Salud de Coclesito, Colón, Panama; borisgarrido1@gmail.com

**Keywords:** clinical manifestations, epidemiological challenges, Panama, *Rickettsia rickettsii*, tick-borne rickettsioses

## Abstract

*Rickettsia rickettsii* is the most virulent agent of the genus *Rickettsia* that causes one of the most relevant vector-borne diseases in the Americas (RRSF). RRSF manifests with many non-specific acute clinical symptoms complicating its diagnosis and can lead to death if not treated appropriately. RRSF has been reported in Canada, the United States of America, Mexico, Costa Rica, Panama, Colombia, Brazil, and Argentina. In addition to *R. rickettsii*, mild and severe spotted fever group rickettsioses (SFGR) have been reported in the Americas; however, the true prevalence of these diseases is unknown. In Panama, RRSF have been reported in four of 14 provinces during two outbreak periods: five cases including two fatalities were identified in 1950–1951, and 23 cases including 17 fatalities between 2004 and 2025. This paper presents the clinical characterization of a fatal case of RRSF in Coclé province and a severe case of SFGR in a mountainous area of the Gnäbe Buglé Indigenous Comarca (GBIC). Laboratory confirmation was performed by molecular analysis of tissues obtained from necropsies in the case of RRSF and by immunofluorescence assay (IFA) in the case of SFGR. Furthermore, this paper identifies existing gaps in the initial clinical suspicion and pertinent to SFGR in Panama, which may be applicable to other countries in the region. In the last 21 years, cases have occurred upon contact with ticks in rural areas (13), urban and suburban locations (7), rural woodlands (2), and forests (1). Provinces with more cases are Panamá (7 of 23, 6 died), Coclé (5 of 23, 5 died), Colón (3 of 23, 1 died), Panamá Oeste (1 of 23, 1 died), and GBIC (7 of 23, 4 died), including a cluster of seven cases in 2019. Therefore, Coclé province is considered one of the endemic areas for RRSF in Panama, while the latest cases from the GBIC since 2019 indicate that mountainous areas are an eco-epidemiological scenario to include in the transmission of these diseases. Although this disease has a low prevalence, patients who present symptoms commonly associated with more common diseases such as dengue, other arboviruses, malaria, and leptospirosis, among others, should be included in the diagnostic suspicion. Without diagnostic suspicion and adequate treatment, the patient can die.

## 1. Introduction

Tick-borne diseases (TBD) include a diverse group of clinical conditions caused by hemoparasites, viruses, and bacteria that affect hundreds of thousands of people worldwide [1,2]. In the Americas, *Rickettsia rickettsii* spotted fever (RRSF) is the most severe of known spotted fever group rickettsioses (SFGR) [3,4,5]. Clinical cases have been reported from Canada, the USA, Mexico, Costa Rica, Panama, Colombia, Brazil, and Argentina; and the disease is considered hyperendemic in some states of Mexico and Brazil [3]. Historically, this disease has been known as Rocky Mountain Spotted Fever, although it has also taken on regional names such as Tobia Fever (Colombia) or Brazilian Spotted Fever (Brazil) [5]. Throughout this distribution, genetic variability has been reported between *R. rickettsii* strains from North America and those from Central and South America, with a higher number of polymorphisms observed in North America [4,5]. This variability may suggest a difference in virulence within each population, and a possible North American origin [5]. In fact, there are avirulent (e.g. Iowa) and virulent (e.g. Shelia Smith) strains in North America, while Brazil, Colombia or Costa Rica only contain virulent strains (e.g. Taiacu); however, this may be a bias because the samples were obtained from patients [5]. Furthermore, the recent descriptions of *R. rickettsii* subspp. *californcia*, and *Rickettsia lanei*, a new species related to *R. rickettsii*, raise new challenges in its taxonomy and in the ecoepidemiology of SFGR in the Americas [6,7], particularly regarding *R. rickettsii* subspp. *Californica*. This subspecies was described from *Dermacentor occidentalis* isolates collected in California, USA [6]. This strain was known as *Rickettsia* species 364-D, *Rickettsia* 364, or *Rickettsia philipii* and causes a moderately severe disease in humans called Pacific Coast Tick Fever. It displays low virulence in guinea pigs and field mice in the laboratory; however, it is lethal in chicken embryos [6]. *Rickettsia lanei*, isolated from *Haemaphysalis leporispalustris* (USA), has demonstrated a close relationship with *R. rickettsii californica* [7].

Clinically, RRSF presents an acute febrile illness with severe headache and maculopapular or petechial rash, and many nonspecific symptoms such as myalgia, arthralgia, headache, dizziness, sore throat, diarrhea, photophobia, dysuria, and conjunctivitis [1,8,9]. Progress of untreated infection causes damage to the endothelium of blood vessels and organs, and can lead to intravascular coagulopathies, or kidney, respiratory, heart, and nerve failure [8,9]. Treatment consists of doxycycline, another type of tetracycline or azithromycin; however, due to the severity of the disease, it is recommended to administer it in the first few days of care [10]. In untreated cases, mortality can be high (20–70%), while patients recovering from severe forms of RRSF infection may experience hearing loss and mental disability, neurological and internal organ damage, or limb amputation [3,8].

Along with RRSF, other SFGR with similar symptoms and mild, moderate, or even severe clinical manifestations also reported in endemic areas for RRSF [11]. In this regard, in the Americas there are reports of rash caused by *Rickettsia amblyommatis* [12], mild fevers with scar by *Rickettsia parkeri* [13,14,15], febrile cases caused by *Rickettsia massilliae* [16], and traveler rickettsiosis caused by *Rickettsia africae* [17,18,19]. Moreover, there are suspicions of RRSF in severe and fatal cases in all Central American countries [20,21]. Since some species of ticks are both vectors and reservoirs of RRSF, knowing the ecology of these vectors is a first step to understanding the epidemiology of these diseases.

As in other countries, RRSF is a disease with a low incidence in Panama, but with a high mortality rate. In the mid-20th century, five cases were reported, with two fatalities (mortality rate of 40%) [10]. According to Calero et al. [10], the recovery of 3 patients was due to the early suspicion of rickettsioses and timely treatment. A second peak of cases occurred from 2004 to 2024; 19laboratory-confirmed RRSF cases were identified, including 17 fatalities (mortality of 89%). In addition, 3 severe non-fatal SFGR cases were diagnosed using serological methods during the same period [3,22,23]. Clinical cases of RRSF have been reported in both rural and urban areas which could lead to a wide spread of this disease.

The objective of this work is to present the clinical and epidemiological aspects of two new cases of SFGR, including another fatal RRSF case in Panama. These findings are presented and discussed in the context of the existing knowledge about the epidemiology of SFGR in Panama with an emphasis on existing gaps and future research directions to address this public health problem.

## 2. Case 1

On 25 July 2024, a 20-year-old male patient from Cutevilla (Llano del Norte, La Pintada, Coclé, Panamá) was presented at Centro de Salud de Coclesito (CSC, Colón, Panamá) with a 2-day history of fever, cephalea, vomiting, nasal congestion, rhinorrhea and hacking cough. On admission, physical evaluation revealed a normal constitution individual weighting 63.9 kg, his body temperature 38.4 °C, a heart rate of 116 bpm, a respiratory rate of 16 rpm, and oximetry with oxygen saturation of 99%. Detailed examination revealed hyaline rhinorrhea and hyperemia with a whitish pharyngotonsillar exudate on oropharyngeal examination and the remaining physical examination was normal. He was initially diagnosed with acute pharyngotonsillitis and prescribed amoxicillin at a dose of 500 mg three times a day for 7 days, along with paracetamol, ibuprofen, dimenhydrinate, and bromhexine, and discharged.

A day later, the patient returned to the same clinic with persistent vomiting and fever. At that time, he was febrile and had stable vital signs. He was prescribed 500 mL of intravenous Ringer’s lactate and routine laboratory tests (complete blood count and urinalysis), as well as dengue tests. He was advised to continue the prescribed medications and return to the clinic with the results of the lab tests prescribed.

On 27 July 2024, the patient returned due to persistent headache, repeated vomiting (3 or more episodes per day), and choluria. The patient denied bleeding or abdominal pain. The physical examination revealed a blood pressure 109/59 mmHg, a body temperature 38 °C, a heart rate 100 beats per min and a respiratory rate 18 breaths per min, and oxygen saturation of 99%. Laboratory results showed a hemoglobin level of 14.0 g/dL, a hematocrit of 42%, a total leukocyte count of 2.7 cells/mm^3^ (with a neutrophil differential of 77% and lymphocytes of 13%), and a platelet count of 49,000/uL. Urinalysis showed amber-colored urine, cloudy appearance, specific gravity of 1.030, pH 5, albumin 2+, erythrocytes 1 × HPF, leukocytes 7 x HPF, a fair number of epithelial cells, urate crystals 2+, bacteria: numerous, bile 2+, urobilinogen 2+, negative occult blood, and negative nitrites. One hour after his admission, the patient was transferred to the Hospital Aquilino Tejeira Hospital (HAT, Penonomé, Coclé province), with a suspicion of dengue fever with warning signs. Immunoserological tests for dengue were IgM negative and IgG positive; an NS1 antigen test was not performed.

In the HAT emergency room, the patient was treated with hydration and admitted to the medical ward on 28 July. He continued to experience osteomyalgia, nausea, and vomiting. He denied bleeding or other symptoms, and his oxygen saturation was 84%. Oxygen was administered at 4 L per min via nasal cannula. On the 29th, he continued to experience desaturation and tachycardia, along with increased abdominal pain.

On 30 July, the patient had a tonic–clonic seizure lasting approximately 2 min with sphincter relaxation. The patient became restless and responded to calls. His pupils were reactive to light; his oxygen saturation was 86% with 10 L per min oxygen supplied by a reservoir mask. He was afebrile, with capillary blood glucose levels 65 mg/dL, and Glasgow 11/15 (eye opening on verbal command, with incomprehensible sounds and motor movements on verbal command). His blood pressure was 140/80 mmHg, and his respiratory rate was 24 breaths per min. Phenytoin 1 gr, SSN, and ½ ampule of diazepam were administered intravenously over 30 min. On re-evaluation, the patient was less restless, saturating at 98%. Blood gas results were consistent with metabolic acidosis with elevated lactate: pH: 6.98, PCO_2_: 27, PO_2_: 51, Lactate: 17.1, HCO_3_: 6.6, Na: 131, K: 4.1. Then HCO3 #3 vials stat, D/SSN 850 mL + HCO3 #3 vials pp IV at 80 cc per h, amiodarone 150 mg IV stat, levofloxacin 750 mg IV stat, and antibiotics Meropenem + Vancomycin IV were administered. On 30 July, a chest X-ray revealed significant bilateral infiltrates. Intravenous furosemide and 150 mg of amiodarone were administered, and blood gases were analyzed after nebulization with Atrovent. *Hantavirus* infection or leptospirosis were suspected based on clinical manifestations. Next day in the morning, the patient went into cardiorespiratory arrest, and CPR was administered, and it was decided to secure the airway for transfer to a hospital with an intensive care unit. Direct laryngoscopy was performed, visualizing the glottis and vocal cords. The patient was intubated with a 7.5 ETT in one attempt, with no complications. The patient suffered three cardiopulmonary arrests for which advanced cardiopulmonary resuscitation was performed, with adrenaline administration. Transfer was arranged to the Rafael Estévez Hospital (Aguadulce, Coclé province), but the patient suffered a fourth cardiopulmonary arrest. After multiple efforts of resuscitation, the patient expired.

### 2.1. Laboratory Investigation

Postmortem samples of kidney, spleen, and liver were sent for diagnosis of *Hantavirus* infection and leptospirosis, which were tested negative. DNA was extracted from necropsy specimens of kidney and spleen using the DNeasy tissue kit (Qiagen, Hilden, Germany), following the manufacturer’s instructions for tissue samples. Extracted DNA was tested by a battery of PCR protocols targeting *Rickettsia* spp. using specific primers and published protocols for *glt*A, *omp*A, and *omp*B genes [23,24]. In all PCR assays, distilled water was used as a negative control, and commercial *Rickettsia conorii* strain (Vircell, Microbiologist) as a positive control. Positive samples were purified using ExoSap (USB) and sequenced with an automated sequencer (Applied Biosystems model ABI Prism 3130 × l Genetic, Foster City, CA, USA). DNA of *R. rickettsii* was detected in kidney and spleen, amplicons had 99% sequences identity to *R. rickettsii* Sheila Smith reference sequences to *gltA* (GenBank CP000848.1), and 100% to *ompA* (GenBank CP121767.1), and *ompB* genes (GenBank CP000848.1). Partial *R. rickettsii* gene sequences generated in this study are deposited in the GenBank database: spleen (*gltA*: PX409978; *ompA*: PX409980; *ompB*: submitted); kidney (*gltA*: PX409977; *ompA*: PX409979; *ompB*: PX409982).

### 2.2. Epidemiological and Ecological Investigation

The patient was a farmer that resided in a rural region on the border between the provinces of Coclé and Colón, at an altitude of approximately 100 m above sea level (masl). The main economic activity in this region is livestock farming, and a copper mine operates in the north of the region. The patient lived with his family (a woman and a 2-year-old child) in a concrete house with a zinc roof, equipped with a kitchen with a stove, electrical and sanitary services, and a shed in the backyard. The owners have two dogs from whom adult ticks were removed, and questing ticks were found on external walls and cement breeze blocks. All ticks were identified *Rhipicephalus sanguineus* s.l. (31 larvae, 18 nymphs, 12 males, 19 females) using the taxonomic criteria of Bermúdez et al. [21]. The patient worked on a farm near the home, where he stayed for several days. It was not possible to visit the area where the patient worked.

A case of RRSF was reported in this area in 2019, and other cases of this disease were reported in 2008 and 2017 in El Macano and El Valle of Anton, Coclé province (Supplementary Material).

## 3. Case 2

On 22 February 2025 a 2-year-old boy from Llano Tugrí (Peña Blanca, Gnäbe Buglé indigenus comarca-GBIC) was admitted to Hospital Materno Infantil “José Domingo De Obaldía” (HMIJDDO), in David city, Chiriqui. This patient was transferred from Hospital Regional Chiriqui Este in San Félix, with a 7-day history of fever, headache, vomiting of food, dysuria, and watery, green, foul-smelling diarrhea, with no mucus, blood, or parasites. His mother reported that for the past day his legs had been “stiff” and that there were “bruises” in the abdominal area; in addition, the child experienced an episode of stiffness with “rolling eyes”, so he was taken to the health center, from where he was transferred to the hospital. The mother stated that the patient had no known medical history and denied respiratory symptoms or mentioned other sick people in the home and did not travel outside his community. The mother stated that the patient had no known medical history and denied respiratory symptoms or mentioned other sick people in the home.

During the consultation, the patient was intubated under sedation, and evidence of hypotension (a blood pressure 79/38 mmHg), cold, marbled skin, multiple petechiae, ecchymoses, and hematomas with a generalized distribution were evident, icteric tinge to the sclera, dry oral mucosa, with signs of bleeding, neck showed bilateral cervical lymphadenopathy, measuring <0.5 cm in diameter. The chest showed petechial lesions and hematomas. In addition, other observations included rhythmic heart, tachycardic, without murmurs or gallop; lung fields were bilaterally rough; abdomen showed purpuric lesions, soft, depressible, without rebound, and without visceromegaly; extremities showed hard edema of 2+, with a generalized petechial rash, predominantly on the thighs, which included involvement of the palms and soles, and capillary refill time was 3 s.

At the time of consultation, the patient presented with a body temperature of 38.8 °C. He was admitted on the same day in the Pediatric Intensive Care Unit (PICU) with diagnoses of septic shock, multiple organ failure, chronic malnutrition; dengue, leptospirosis, malaria, hemolytic uremic syndrome, Reye’s syndrome, and cholestatic syndrome were considered for differential diagnosis. The admission blood count revealed mild leukocytosis (13.5 × 10^3^ cells/uL), moderate anemia (7.3 g/dL), and severe thrombocytopenia (9 × 10^3^ cells/uL). Elevated transaminases alanine aminotransferase (ALT) at 68 U/L and aspartate aminotransferase (AST) at 190 U/L were observed. Cerebrospinal fluid showed no evidence of changes suggestive of infection. Imaging studies revealed no infiltrates or consolidations in chest X-ray. An abdominal ultrasound detected intestinal parasites and scant fluid in the flank and right iliac fossa. Retroperitoneal lymph nodes were not enlarged. Finally, a brain computed tomography scan revealed no signs of pathology, and an electroencephalogram showed no paroxysmal activity.

The patient was managed with advanced life support, antipyretics (acetaminophen), anticonvulsants (phenytoin, levetiracetam), antibiotic coverage (Ampicilina-sulbactam 450 mg iv c/6 h, Metronidazol 90 mg iv c/day, Cefepime 450 mg iv c/8 h, Cefotaxima 435 mg iv c/6 h, Clindamycin 120 mg iv c/8 h, Meropenem 370 mg iv c/8 h), as well as transfusions of cryoprecipitate, fresh frozen plasma, and packed red blood cells, and supportive care. Multiple platelet transfusions were required. Doxycycline 25 mg by nasogastric tube c/12 h treatment began on 24 February 2025 (2 days after admission). After sedation levels were lowered, a scheduled extubating was performed starting on 2 March 2025 (8 days after admission). The patient had a favorable clinical course, without seizures or fever, and was extubated on his ninth hospital day. He remained in the PICU for a total of 10 days and was subsequently transferred to the pediatric ward, where he completed the course of antibiotics and was assessed for his nutritional status until his discharge after 18 hospital days.

### 3.1. Laboratory Investigation

Samples to rule out dengue, Zika, Chikungunya, malaria, SARS-CoV-2, parvovirus B19, leptospirosis, viral hepatitis, cytomegalovirus, and Epstein–Barr virus infection were processed by the hospital laboratory; all tests were negative. To rule out rickettsial infection, immunofluorescence assay (IFA) for TGR and SFRG was performed, following the recommendations of the Focus Diagnostics^®^ kit manufacturer (Cypress, CA, USA) that utilizes inactivated *R. rickettsii* and *R. typhi* antigens. Additionally, blood DNA extraction and PCR tests similar to those described in case 1 were performed. The results were positive for IgM-SFRG (IFA titer 1:256) and negative for IgM-TGR. No *Rickettsia* DNA was detected in the blood sample collected upon arrival at the hospital. On 10 March 2025, the patient blood was re-evaluated by IFA, resulting in 1/256 IgG antibody titter to SFRG.

### 3.2. Epidemiological and Ecological Investigation

The patient resides in a rural, mountainous region in GBIC, at an altitude of approximately 1700 masl. This area is connected by road to the Inter-American Highway. The patient’s home was evaluated by the Vector Control Unit, revealing block construction, a tile floor, a zinc roof, a stove-type kitchen, solar panel lighting, a septic tank, and waste management by incineration. The home is inhabited by 10 children and 5 adults. The father works on a coffee farm outside the comarca and had not been in contact with him or reported traveling outside their community for a month prior to the onset of symptoms, and his mother is a homemaker. No information was provided regarding the background of the tick bites. The owners have chickens and three dogs.

Adult and immature ticks were removed from the dogs, and identified as *Ixodes* cf. *boliviensis*, *Amblyomma ovale*, and *Amblyomma mixtum* using taxonomic criteria for adults [25]. *A. mixtum* nymphs were identified by molecular methods [26]. Ticks were analyzed following the method described above for the detection of *Rickettsia*. All ticks were tested negative for *R. rickettsii* DNA. This is the second episode of RRSF in GBIC, since the first outbreak occurred in 2019, when 6 people were affected and 4 died. This region is located approximately 940 m above sea level and lacks land routes (Supplementary Material).

## 4. Epidemiological Review

To understand the epidemiology in Panama, a review of reported and published cases was conducted. Data of the confirmed and reported cases in Panama are shown in Supplementary Material.

## 5. Discussion

### 5.1. Clinical Features

During the initial evaluation in both cases, the patients presented nonspecific symptoms such as fever, headache, vomiting, and gastrointestinal discomfort. In addition to these symptoms, the appearance of a skin rash in case 2 raised the diagnostic suspicion for RRSF. In both patients, the symptoms worsened as the disease progressed resulting in seizures and tachycardia. The initial suspicion in case 1 was a pharyngeal infection and dengue, another arboviral infection like Zika, Chikungunya or Oropuche virus, leptospirosis and *Hantavirus* infection in both cases. These diseases are more prevalent in Panama; however, they were ruled out upon negative laboratory testing. These patients developed thrombocytopenia and leukocyte abnormalities, leukopenia (case 1) and leukocytosis (case 2), consistent with acute rickettsioses but not common for other diseases [27,28,29,30]. Furthermore, the elevated liver enzymes, metabolic acidosis, and pulmonary infiltrates seen in case 1 are consistent with the development of multiorgan failure and interstitial pneumonitis, and are signs of systemic damage and central nervous system disorders that provoked shock and death [8,27,29,30,31]. The clinical description of cases in Panama was recently compiled [31].

One aspect that stands out in these cases is that none of the cases reported or indicated contact with ticks, and in case 1 no petechiae or rash were reported. Regarding these, about 10% of patients do not develop petechiae or rash, and they are hardly distinguishable in dark-skinned people [32,33,34]. The lack of a history of tick bites or the absence of petechiae cannot be a determining factor for including clinical suspicion and initiating treatment, since the patients with RRSF deteriorate rapidly, and the fatal cases are associated with lack of treatment with doxycycline or with untimely treatment.

According to Foley et al. [3] most fatal cases of RRSF occur within the first 8 days of infection, providing a narrow therapeutic window. Some authors show that children, older adults, or people with comorbidities are among the highest risk groups [35], therefore the initiation of treatment must be based on clinical suspicion, as in case 2 that had a satisfactory outcome due to the treatment [10], which contrasts with the other case where the patient died [24,27,36], even with doxycycline treatment [37]. Although there is a guideline for the treatment of rickettsiosis in Panama [38] and there are epidemiological data, clinical suspicion has not yet been established in many regions of the country. Cases not diagnosed in time may be considered medical negligence in this country [39].

Since 2004, diagnosis has been based on serology by IFA, PCR, and sequencing and isolation (Supplementary Material). IFA only allows the diagnosis of SFGR if there is evidence of IgM-IgG seroconversion in samples taken in the acute and convalescence phases but does not allow to determinate the *Rickettsia* species unless multiple antigens are tested side-by-side and cross-absorption is performed to determine the etiological agent. In contrast, use of PCR and amplicon sequencing of *R. rickettsii* DNA from blood and necropsy permitted reliable confirmation of RRSF in Panama (Supplementary Material).

### 5.2. Epidemiological Challenges

Compared to other vector-borne diseases, the ecology of RRSF is complex and among the least understood, especially in Central America [24]. This disease has been reported in different types of ecosystems from southern Canada to northern Argentina, involving several vector tick species, including endemic (*Amblyomma* and *Dermacentor*) and species introduced during the European conquest in America (*Rhipicephalus sanguineus* complex) (Table 1), in addition to an undetermined number of amplifying reservoirs that vary according to region. Risk factors for RRSF are related to exposure to infected ticks; hence, individuals who engage in outdoor activities or have pets are most vulnerable [34,40,41,42]. Since some tick species serve as both vectors and reservoirs of *R. rickettsii*, knowing their ecology, phenology, and abundance is the first line of information needed to assess the risk of exposure in different parts of the country [41,43]. A second line of information may be the presence of vertebrates that serve as amplifier reservoirs to *R. rickettsii*, information which is not available in most countries [41,42]. Thus, gaps of biological information constitute a challenge in many countries where updated tick lists are unavailable or where competent vectors have not been studied in the laboratory. However, the lack of this type of data should not diminish diagnostic suspicion in countries where cases have occurred.

In Panamá, the epidemiology of RRSF is not fully understood. In the last 21 years, cases have occurred upon contact with ticks in rural areas (13), urban and suburban locations (7), rural woodlands (2), and forests (1). Provinces with more cases are Panamá (with a family cluster of three cases), and Coclé and Colón, Panamá Oeste and GBIC, including a cluster of seven cases in 2019. Farmers and forest workers appear to have the highest risk of tick encounters; however, numbers of cases are linked to tick exposure in peridomestic rural and urban environments. The age of individuals diagnosed with RMSF in the last 20 years ranges from 2 years old to 60 years old, and most cases occur locally.

The first national serological survey in Panama was conducted in the mid-1950s, demonstrating a seropositivity rate of between 5% and 15% in a sample of 1400 volunteers [59]. In 2013, a seroprevalence study was conducted among people who handled wild and domestic animals, of 97 healthy volunteers from the communities of Tortí (Darién province), Parque Municipal Summit (Panama province), and El Valle de Antón (Coclé province), 38 (39%) IgG-type antibodies to SFGR using inactivated *R. rickettsii* and *R. typhi* antigens [60]. An animal survey detected IgG antibodies reacting to *R. rickettsii* antigen in dogs and horses from El Valle de Antón [61], and synanthropic mammals as such opossums and a coyote in Panama and Panama Oeste provinces [62]. Several species and unnamed isolates of SFGR have been identified in many tick species collected in Panama [2]. These data demonstrate the circulation of SFGR among ticks and animals in several locations in Panama and that people have contacts with *Rickettsia*-infected ticks, including areas where cases have been reported. A molecular survey of wildlife and peridomestic animals should be conducted to determine the circulation of *R. rickettsii* or to establish which animals could be amplifying reservoirs.

*Amblyoma mixtum* and *R. sanguineus* s.l. are viewed as most likely vectors of *R. rickettsii* in Panama—both ticks have a broad area of distribution across the country [10,24,53,54,63,64]; however, their vector competence is not fully evaluated. *Amblyomma mixtum* is found mainly in lowlands (below 1000 masl) in the Pacific Basin, persisting in grazing areas, riparian, and secondary forests; *R. sanguineus* s.l. is an intra-domiciliary tick species found in urban, suburban, and rural dwellings [54,64,65]. The distribution areas of both ticks overlap with the geography of 47% of RRSF and SFGR cases, which exclude those from wild areas (absence of *R. sanguineus* s.l.) and urban (absence of *A. mixtum*). Moreover, tick distribution overlaps with the distribution of vectors of more prevalent pathogens, especially *Aedes* and other mosquitoes (urban and rural environments), and with diseases such as leptospirosis (rural environments), in addition to other pathologies such as SARS-CoV-2, parvovirus B19, viral hepatitis, and cytomegalovirus [66,67,68,69].

The majority of RRSF cases in Panama occurring in rural settings are concordant with the presence of *A. mixtum*, which is an eclectic species [54,64]. RRSF eco-epidemiology associated with *Amblyomma cajennense* complex ticks is complex, as it involves multiple species of potential amplifying reservoirs. In Brazil, *Amblyomma sculptum*, another species of the *A. cajennense* complex, parasitizes several species of mammals and it has been recognized that the capybara (*Hydrochoerus hydrochaeris*) serves as amplifying reservoirs of *R. rickettsii* [41]. Although in Panama the related species *Hydrochoerus isthmius* is a host of *A. mixtum* [70], its susceptibility to *R. rickettsii* has not been studied; however, this rodent species only inhabits the eastern portion of Panama and, to date, no cases have been confirmed in that region. This represents that most cases that are assumed to be related to *A. mixtum* must have another mammal species as a reservoir. Although horses are common hosts of *A. mixtum* in Panama [69,70], their role as amplifiers of *R. rickettsii* has not been demonstrated. Another scenario would suggest that it may be associated with *R. sanguineus* s.l. infesting homes in urbanized settlements, with dogs as putative reservoirs. Since *R. rickettsii* can cause disease in dogs [47], it is necessary to establish measures to separate its symptoms from those of canine ehrlichiosis, another disease transmitted by *R. sanguineus* s.l.

Regarding the current cases, despite *R. rickettsii* not being detected in the ticks analyzed from both cases, the presence of *A. mixtum* in GBIC at 1700 masl and in a locality of SFGR could indicate a new ecological scenario for these diseases, associated with a more temperate climate. In 2019, an RRSF family cluster moved to another town in GBIC. Given this, more data is needed to confirm vectors in regional areas, particularly because the patient is a young child and *A. mixtum* is not an intra-domiciliary species. Even so, the existence of foreign species such as *Dermacentor andersonii* or *Dermacentor variabilis* can be ruled out as a vector in Panama [71]. The failure to find *R. rickettsii* in ticks is not unusual, as low infection rates have been reported in tick populations in RRSF-endemic areas [72]. However, unlike the present case, the majority were people who were able to travel from other locations, while the patient’s mother reported no movement prior to the onset of symptoms.

## 6. Conclusions

RRSF is an epidemiologically complex disease that puts its sufferers at risk of death; therefore, early diagnostic suspicion is required to enable appropriate treatment within the first few days after the onset of symptoms. Even when this disease has a low prevalence, and epidemiological information is lacking, it should be included in the diagnostic suspicion for patients presenting with fever, malaise, thrombocytopenia, leukopenia, or altered liver enzyme levels, which suggest diseases that have a higher incidence, such as dengue and other arboviral infections, malaria, and leptospirosis, among others. In Panama and other countries in the region, studies should be established that integrate the distribution and ecology of its potential vectors to develop specific prevention programs in endemic regions, especially considering that the ecoepidemiology of SFGR varies from one region to another [52]. Finally, future research could evaluate the genetic variability of *R. rickettsii* in Central America, especially considering the recent descriptions of *R. lanei* and *R. rickettsii californica* [6,7,52].

## Figures and Tables

**Table 1:** Ticks related to *Rickettsia rickettsii* according to vector competence or natural infection.

Competent Vectors ^a^	Country	Genus	Species	References
	Argentina	*Amblyomma*	*tonelliae*	[44]
	Brazil	*Amblyomma*	*dubitatum*	[45]
	Brazil	*Amblyomma*	*sculptum*	[46]
	Brazil	*Rhipicephalus*	*sanguineus* s.l.	[47]
	Canada	*Dermacentor*	*andersonii*	[48]
	Canada	*Dermacentor*	*variabilis*	[48]
	Colombia	*Amblyomma*	*patinoi*	[49]
	Mexico	*Rhipicephalus*	*sanguineus* s.s.	[50]
	USA	*Amblyomma*	*americanum*	[51]
	USA	*Dermacentor*	*andersonii*	[48]
	USA	*Dermacentor*	*variabilis*	[48]
	USA	*Dermacentor*	*occidentalis*	[52]
Potential vectors ^b^	Panama	*Amblyomma*	*mixtum*	[53,54]
	Panama	*Rhipicephalus*	*sanguineus* s.l.	[10,24]
No evidence of vector capacity ^c^	Mexico	*Amblyomma*	*americanum*	[55]
	Mexico	*Amblyomma*	*maculatum*	[55]
	Mexico	*Amblyomma*	cf. parvum	[55]
	Mexico	*Amblyomma*	*tenellum*	[55]
	Mexico	*Dermacentor*	*nitens*	[55]
	Mexico	*Ornithodoros*	*nicollei*	[55]
	Mexico	*Otobius*	*lagophilus*	[55]
	Costa Rica	*Haemaphysalis*	*leporispalustris*	[56]
	Costa Rica	*Amblyomma*	*varium*	[57]
	Costa Rica	*Amblyomma*	*mixtum*	[57]
	Panama	*Dermacentor*	*nitens*	[58]

^a^ Vectorial competence demonstrated under laboratory experiments. ^b^ Suspected vectors by implication in case, but without laboratory experiments. ^c^ Natural infections.

## Data Availability

The original contributions presented in this study are included in the article/Supplementary Material. Further inquiries can be directed to the corresponding authors.

## Supplementary Materials

The following supporting information can be downloaded at: https://www.mdpi.com/article/10.3390/pathogens14101006/s1, Table S1: Demographic data of SFGR and RRSF in Panama (1950–1951; 2004–2025).

## Abbreviations

The following abbreviations are used in this manuscript:

CSC	Centro de Salud de Coclesito
GBIC	Gnäbe Buglé Indigenous Comarca
HAT	Hospital Aquilino Tejera
HMIJDDO	Hospital Materno Infantil “José Domingo De Obaldía”
IFA	Immunofluorescence Assay
SFGR	Spotted Fever Group *Rickettsia*
RRSF	*Rickettsia rickettsii* Spotted Fever
TBD	Tick-borne Disease
